# Psychological Capital Profiles and Their Relationship With Internal Learning in Teams of Undergraduate Students

**DOI:** 10.3389/fpsyg.2022.776839

**Published:** 2022-01-20

**Authors:** Rosa Lutete Geremias, Miguel Pereira Lopes, André Escórcio Soares

**Affiliations:** ^1^Centre for Public Administration and Public Policies, Institute of Social and Political Sciences, Universidade de Lisboa, Lisbon, Portugal; ^2^Faculty of Health and Life Sciences, Coventry University, Coventry, United Kingdom; ^3^Faculty of Economic Sciences and Management, Nicolaus Copernicus University in Toruń, Toruń, Poland

**Keywords:** psychological capital profiles, self-efficacy, hope, optimism, resilience, internal learning in teams, undergraduate students

## Abstract

This study aims to analyze the relationship between psychological capital profiles and internal learning in teams. The participants in this study were 480 undergraduate students. We performed a cluster analysis using the SPSS and yielded four distinct psychological capital profiles. The student profile with the highest scores in self-efficacy, optimism, hope, and resilience (Profile 2-Fully PsyCap) exhibited also the highest scores of internal learning in teams. On the other hand, the student profile with the lowest scores in self-efficacy, optimism, hope, and resilience (Profile 1- Empty PsyCap) presented the lowest scores of internal learning in teams. It is also noteworthy that there was no significant relationship between the profile with a positive combination between self-efficacy and hope (profile 4) and the profile that presents the optimism as the only positive psychological capability (profile 3), in the way they relate to internal learning in teams, which led us to reject the second hypothesis of the study. This study reinforces the role of psychological capital in academic settings and suggests that psychological capital profiles can affect internal learning in teams differentially.

## Introduction

A review of the literature on internal learning in teams highlights the need to analyze the circumstances that can drive learning (Druskat and Kayes, 2000; Bresman and Zellmer-Bruhn, 2013; Vignery and Laurier, 2020). Thus, identifying the antecedents that influence the variation in the internal learning in teams process allows us to understand why some students are not successful (Cheryl et al., 2007; Martínez et al., 2019; Geremias et al., 2020).

Academic performance studies point out to the analysis of internal learning in teams as a top priority for students, school and university administrators (You, 2016; Soares and Lopes, 2017; Datu and Valdez, 2019). However, traditional predictors of internal learning in teams have focused on analyzing differences in learning rates obtained through standardized aptitude tests, leaving aside other important psychological and structural factors that can influence the academic success (Cavanagh, 2011; Dickerson et al., 2013; Geremias et al., 2021).

While broadly ignored in educational settings, Vignery and Laurier (2020) have found that psychological capital (PsyCap) can be considered a potential factor underlying academic success. Psychological capital (PsyCap) emerged from the positive psychology movement and has been related to different attitudes, behaviors, and performance of employees in the workplace (Avey, 2014; Newman et al., 2014; Luthans and Youssef-Morgan, 2017). From this perspective, PsyCap is a second-order construct defined as the positive psychological state of development of an individual, consisting of four sub-dimensions: self-efficacy, optimism, hope, and resilience (Luthans et al., 2007, 2010; Ferradás et al., 2019).

Different empirical studies pointed out the psychological capital as a second-order construct, due to the positive interaction between the four psychological capabilities (Roche et al., 2014; Daspit et al., 2015; Friend et al., 2016). Although the PsyCap sub-dimensions are correlated, certain studies, for example, Luthans et al. (2007), have shown that empirically they are independent and have discriminating validity, so people can have different levels of self-efficacy, hope, optimism, and resilience. Dawkins et al. (2013) argued that PsyCap studies have been neglecting the importance of examining an individuals’ Psycap profile. Moreover, it may be interesting to see if PsyCap profiles can differentially affect the results (Luthans and Youssef-Morgan, 2017). In terms of an individuals’ Psycap profile, empirical evidence confirmed the existence of teachers profiles that differ in their psychological capital (Ferradás et al., 2019).

These identified PsyCap profiles were related to the results of performance and employee satisfaction variables, such as burnout (Bouckenooghe et al., 2018; Djourova et al., 2019; Ferradás et al., 2019). However, too often it has been overlooked that university students also face different challenges, such as uncertainties of an ever-changing economy (Siu et al., 2013; Soares and Lopes, 2017). Hence the need to conduct studies that contribute to building positive psychological resources for students to address underperformance or identify learning disabilities (Luthans et al., 2012). For Luthans and Youssef-Morgan (2017) there have been only a few attempts to conduct studies that allow us to examine whether the effects on internal learning in teams differ depending on the different combinations of the four sub-dimensions of PsyCap.

Our logic here is based on research suggesting that different combinations of the four sub-dimensions of PsyCap may affect outcomes differently (Bouckenooghe et al., 2018; Djourova et al., 2019). With these points in mind, this study aims to analyze the relationship between psychological capital profiles and internal learning in teams, as the relationship between psychological capital and internal learning in teams, has already been empirically demonstrated (Geremias et al., 2021). By doing so, it contributes to the study of the relationship between psychological capital and internal learning by exploring the influence of PsyCap profiles on internal learning in teams. Furthermore, Datu and Valdez (2019) mentioned that it is important that the student is committed to promoting internal learning that allows overcoming barriers to academic success. Therefore, for this, he/she needs to develop certain psychological skills.

This study makes important contributions. First, we argue that the present study, when defining psychological capital profiles, can have important implications for the academic field. For example, the study of PsyCap profiles might be a field of intervention and action when students, teachers, and policymakers are particularly interested in developing learning and improving academic success (Geremias et al., 2021). Second, the study of PsyCap profiles offers an opportunity to analyze whether psychological capabilities (self-efficacy, optimism, hope, and resilience) have different levels and may influence internal learning in teams (Luthans and Youssef-Morgan, 2017). As such, this study is in line with recent calls toward analyzing whether psychological capital profiles can differentially affect results (Dawkins et al., 2013; Ferradás et al., 2019).

This paper is organized as follows. First, a review of the literature on Psychological capital profiles and internal learning as well as the outline of the hypotheses of the study. Second, the presentation of the methodological options and procedures and the description of the results. Third, the discussion of the results and outline of the main implications of this study for theory and practice. The paper ends with some concluding remarks.

## Literature Review and Hypotheses

### Psychological Capital: Definitions and Profiles

Psychological capital (PsyCap) reflects an “individual’s positive psychological state of development,” and has been broadly characterized by the psychological resources of self-efficacy, hope, optimism, and resilience (Luthans et al., 2007; Avey et al., 2009; Dawkins et al., 2018). Self-efficacy represents the positive beliefs and thoughts, about one’s personal capabilities to achieve success in challenging tasks (Liao and Liu, 2016); hope is the sense of agency that individuals can achieve their goals and have determined alternative pathways to accomplish defined goals (Snyder et al., 2002; Harms et al., 2018); optimism consists of fostering positive global expectations of success (Ertosun et al., 2015); resilience is the positive psychological capability that allows individuals to face or recover positively from adversity, uncertainty, risk, or failure (Luthans et al., 2014).

The combination of the four psychological capabilities (self-efficacy, optimism, hope, and resilience) provides a high level of psychological capital that allows an individual to focus on performing tasks and having success in completing these tasks (Peterson et al., 2011; Parent-Rocheleau et al., 2020). However, there is empirical evidence that individuals score differently across these four capabilities, for example, score high on some capabilities and low on others (Djourova et al., 2019; Ferradás et al., 2019).

The variation in individuals’ scores across the four psychological capabilities shows that despite being related the different capabilities can be viewed as distinct dimensions (Bouckenooghe et al., 2018). On the other hand, authors such as Djourova et al. (2019) criticized the use of multidimensional constructs, arguing that variations in the overall construct result from variations within one or more of the sub-dimensions that are not explored and, therefore remain obscure.

The few studies on the psychological capital profiles have focused on employee samples, and there is a certain diversity in the profiles identified. Bouckenooghe et al. (2018), identified six profiles exhibited significant quantitative and qualitative differences. First profile (low resilience; high optimism, hope and, self-efficacy); second profile (low optimism; high resilience, hope, and, self-efficacy) and the remaining profiles combined the four components at similar levels— low, moderate, moderately high and high.

Other authors (e.g., Djourova et al., 2019), identified four psychological capital profiles. First profile (low hope and self-efficacy; high in resilience and optimism); second profile (high hope and self-efficacy; low in resilience and optimism); third profile (low self-efficacy; high hope, resilience, and optimism); fourth profile characterized by high levels of the four psychological capabilities. Most of the research on psychological capital profiles has been conducted in the organizational context, however, some authors make a call for these profiles to be studied in other contexts (e.g., Ferradás et al., 2019).

Higher education is generally responsible for educating and preparing students to meet organizational and social demands, so great attention should be paid to student PsyCap (You, 2016). Previous studies have shown that psychological capital is positively correlated to learning engagement (Carmona–Halty et al., 2018); learning empowerment (Datu et al., 2016) and academic performance (Datu and Valdez, 2019). Overall, there is evidence showing that psychological capital is an important predictor of learning and academic success (Lin, 2020).

### Internal Learning in Teams

Internal learning in teams is a process that requires team members to actively and openly question themselves, discuss mistakes and collectively reflect on how the team can achieve its goals and improve performance (Bresman and Zellmer-Bruhn, 2013; Geremias et al., 2020). This process starts with the sharing of knowledge by individuals, through the presentation of ideas among team members, in order to contribute to the development of new perspectives (Kessler et al., 2000; Alyahyan and Düştegör, 2020). Normally, team members actively interact by asking questions about different ideas, actions and communicating openly about errors, which improves the future performance of the team (Edmondson, 1999). Hence, internal learning theorists carry the assumption that team members might generate different approaches to complete new and challenging tasks (Bresman and Zellmer-Bruhn, 2013). This implies that learning in teams requires a discussion of different information and sometimes about uncomfortable topics (Druskat and Kayes, 2000).

Internal learning requires team members to deal with challenging tasks, develop cognitive strategies to achieve success (Pekrun, 1992). Therefore, internal learning allows team members to create alternatives to solve the problems identified in to achieve the defined goals (Chan et al., 2003; Geremias et al., 2021). Teams need to be able to develop practices and procedures that incorporate learning behaviors, and to detect problems in the execution of tasks, as well as to make operational adjustments (Argote and Miron-Spektor, 2011). Thus, teams should encourage the dynamic involvement of their members in collaborative learning processes to create a favorable working climate that contributes to the improvement of individual performance (Song et al., 2014).

In the academic field, learning in teams has been stimulated to contribute to the development of practical knowledge preparing students for the challenging workplace (Cheryl et al., 2007; Park et al., 2015). The ability to work effectively in a team environment within the classroom contributes to academic performance (Soares and Lopes, 2017) and might be considered an essential competence in the workplace (Brown and Latham, 2002). The creation of student teams is crucial to develop teamwork skills through the exchange of experiences and knowledge sharing in order to improve the level of learning about the content of the tasks in progress (Druskat and Kayes, 2000; İlçin et al., 2018).

When students work together in teams, they earn rewards that can be individual as well as collective. Hollifield (1984), emphasized that for achieving an academic goal, all members need to work together for the teams’ success, therefore the team members should encourage and help each other to learn. Gast et al. (2017), reported the success achieved by teams that carry out periodic work with specific tasks, in order to achieve satisfactory results for the whole team.

The learning in teams happens when students realize that they share similar goals. This positive interdependence might allow students to encourage and help each other to achieve the team’s goals (Dickerson et al., 2013; Herrmann, 2013). Learning in teams allows students to achieve positive results, such as collaboration in the development of content, the opportunity to develop critical skills in the interpretation of tasks and a growing awareness of the cognitive processes necessary to achieve goals (Finlay and Faulkner, 2005). There are also social motivations and results of learning in teams, such as interactions with colleagues and the development of communication skills (Cavanagh, 2011; Hayat et al., 2020). Therefore, our contention is that learning in teams contributes and helps students to keep focusing on accurate goals to complete the course successfully.

### Psychological Capital Profiles and Internal Learning in Teams

All four PsyCap capabilities are theorized as resources that strengthen learning, allowing students to overcome uncertainty related to academic success and facilitate future goals attainment (You, 2016; Geremias et al., 2020). However, previous empirical studies found that the four PsyCap capabilities (hope, self-efficacy, resilience, and optimism) might not make equivalent contributions to explaining a specific behavior or outcome (Madrid et al., 2017; Yu et al., 2019).

Different studies found that individuals with high hope have a strong involvement in the internal learning process – compared to those who are low hope (Dixson et al., 2017). This seems to happen because some plans may not be successful. Therefore, high-hope individuals, as measured by having an above-average level of hope, produce multiple alternative paths to circumvent possible obstacles, considering that they are not only better at predicting plausible paths to their goals but, also produce several alternative paths, in case of unexpected challenges or when the defined goals prove unattainable (Feldman and Kubota, 2015).

Individuals with high self-efficacy might pursue successful performance in academics settings, although the strength of relationships varies between studies (Lane and Lane, 2001). Doménech-Betoret et al. (2017) argued that high self-efficacy students display better learning and academic performance, compared to those who are low self-efficacy. Because, students with low self-efficacy have difficulties in performing tasks and might take the assignments harder then what they truly are which results in a limited view on problem-solving and affect negatively the learning outcomes (Motlagh et al., 2011).

Students with high resilience might achieve positive learning outcomes, motivated by the capacity to overcome constant challenges to complete their studies (Brewer et al., 2019). Cassidy (2015) analyzed studies on resilience in academic settings and identified significant differences between resilient and non-resilient students. This author argued that resilient students are more likely to be successful in completing assignments, thus decreasing the risk of failure. Similarly, Martin and Marsh (2009) analyzed students’ profiles according to academic resilience. The results revealed that high resilient students were more persistent, demonstrate more capability to effectively deal with challenges, adversity and low difficulties in accomplishments of tasks.

Previous studies demonstrate that high optimism promotes learning and educational success (Haynes et al., 2006; Tetzner and Becker, 2017). This is because high optimism may contribute to improving students’ academic performance, giving them greater persistence and engagement in carrying out tasks, which may lead them to take active measures to ensure academic success in the future, for example, taking private lessons after failures. Moreover, high optimism influences positively academic results, on the other hand, low optimism has a negative correlation with results in different domains, including learning performance (Harpaz-Itay and Kaniel, 2012). Ortega-Maldonado and Salanova (2017) argued that undergraduate students need high self-efficacy to exert the effort required to complete assignments. Additionally, high optimism helps students to make positive attribution about succeeding.

Hope and resilience are important psychological capabilities, as they allow students to persevere in achieving goals and to deal positively with problems and adversities. On the other hand, self-efficacy and optimism were identified as predictors of academic success in university students (Feldman and Kubota, 2015). For Datu et al. (2016), the combination of the four psychological capabilities may positively influence students’ academic results. Therefore, research on psychological capital profiles (e.g., Djourova et al., 2019), suggests that profile groups with high self-efficacy, optimism, hope, and resilience may be related to positive outcomes in different contexts. By contrast, low self-efficacy, optimism, hope, and resilience may negatively influence academic outcomes (Chen et al., 2019).

Therefore, it is worthwhile to investigate the influence of psychological capital profiles based on the combination of high self-efficacy, optimism, hope, and resilience on internal learning in teams. Alternatively, it is expected that PsyCap profiles with a combination of low self-efficacy, optimism, hope, and resilience will show low scores on internal learning in teams. Given this kind of previous conclusions, we hypothesize that:

*H1: Profile groups with high self-efficacy, optimism, hope, and resilience will have higher scores, regarding internal learning in teams than profile groups with low self-efficacy, optimism, hope, and resilience*.

We also theorize that positive psychological capabilities interact synergistically and may positively influence internal learning in teams. Luthans et al. (2007), noted that positive psychological capabilities may affect individuals’ behavior differently. Research studies in educational settings have shown that students with higher levels of resilience and self-efficacy have stronger academic outcomes (Luthans et al., 2012). For Luthans and Avolio (2014), positive psychological capabilities such as hope and optimism aimed at learning may play a key role, especially when the environment is less favorable to the learning process. On the other hand, Youssef and Luthans (2007), argued that hope, optimism, and resilience are the three positive psychological capabilities that share self-directed motivating processes with a positive impact on individuals’ outcomes.

As acknowledged by Djourova et al. (2019), the dimensions of the PsyCap encompass different elements that may be independent enough that the individual may have high levels of one or two psychological capabilities but low levels of the rest. The study by Dawkins et al. (2013) demonstrate the possibility that a profile with a strong combination of self-efficacy and hope also has a greater influence on different results. Additionally, a recent study highlighted the role of the combination of self-efficacy and hope in explaining academic adjustment (Zhou and Kam, 2016). Furthermore, according to Tomás et al. (2019), the positive combination of hope and self-efficacy may be considered an indicator of academic success. For these reasons, it is expected that profile groups with a positive combination of self-efficacy and hope will have higher levels of internal learning in teams. Given this kind of previous conclusions, we hypothesize that:

*H2: Profile groups with a positive combination of self-efficacy and hope will have higher levels of internal learning in teams than profile groups with a positive combination of other psychological capabilities.*

## Materials and Methods

### Participants and Procedure

We collected data from 480 undergraduate students at different higher education institutions in Angola, including public institutions (81 percent) and private institutions (19 percent). The data collection has been authorized by the board of each institution, and permission has been granted by the lecturer of the modules in which the survey was carried out. Additionally, we collect written informed consent from participants. Students answered the questionnaire in person and voluntarily during the class period, using paper and pencil. They first filled out a questionnaire measuring PsyCap, and then a questionnaire measuring internal team learning was administered.

To ensure confidentiality, the first author distributed and received all the questionnaires in person, and also took the time to clarify any doubts that arose during the entire process. In addition, it was possible to inform all participants that participation was voluntary and the data collected would be processed exclusively by the researchers involved in this study.

In total 600 surveys were distributed to students, and we received 480 valid questionnaires (from 22 classes, ranging from 19 to 44 students per class), which represents a response rate of 80 percent. Overall, 54 percent of the study participants were men, and the average age was 24 years (*SD* = 5.94). The students were from, first-year (61 percent), second-year (21 percent), third-year (11 percent), and fourth-year (7 percent). Moreover, the most significant courses were Economics (25 percent), Business Management (12 percent), Nursing (11 percent), and Linguistics-English (8 percent). On the other hand, 64 percent of participants study in the morning, 2 percent in the afternoon and 34 percent in the after-work period.

### Measures

#### Psychological Capital

We used the version of the 24-item questionnaire adapted for research in the educational context by Luthans et al. (2012). The scale evaluating the four positive psychological capabilities: Self-efficacy (six items; e.g., “I feel confident when I look for a solution to a long-term problem.”); hope (six items; e.g., “There are lots of ways around any problem concerning my schoolwork”); resilience (six items; e.g., “I usually manage difficulties one way or another concerning my schoolwork”); optimism (six items; e.g., “In studies, I am optimistic about what will happen in the future”). The responses were given on a 6-point Likert scale, from (1) “Totally Disagree” to (6) “Totally Agree.” According to Luthans et al. (2012), the 24-item positive psychological capital scale presented in the original study has a Cronbach’s αs of 0.90. The four positive psychological capabilities demonstrated appropriate internal consistency (self-efficacy: Cronbach’s αs of 0.85; hope: Cronbach’s αs of 0.80; resilience: Cronbach’s αs of 0.72; optimism: Cronbach’s αs of 0.79).

#### Internal Learning in Teams

We used the scale adapted by Bresman and Zellmer-Bruhn (2013). This 7-item questionnaire evaluates internal learning. As an example of items is “We regularly reserve time to find ways to improve the group’s work processes.” We used a Likert-type response scale with scores ranging from (1) “Totally Disagree” to (7) “Totally Agree” with a Cronbach’s αs of 0.71. The scales were translated into Portuguese using the translation/retroversion method. The original scale and translated versions were carefully compared, at this stage an English-speaking native and Portuguese–English linguistic lecturer assisted us in this process.

### Measure Validity

The confirmatory factor analysis, carried out using the software Amos v.25 on the scale of psychological capital as a second-order factor, resulted in adequate values. The model presents moderate and good factorial weights (λ ≥ 0.30) and appropriate individual reliabilities (*r2* ≥ 0.10). The final model has excellent adjustment indexes, χ^2^(145) = 242.993, ρ < 0.001; TLI = 0.908; CFI = 0.922; GFI = 0.949; SRMR = 0.044; RMSEA = 0.038. The Cronbach Alpha for the positive psychological capital dimension was 0.86. It is important to emphasize that although we use PsyCap as a second-order factor, to assess the construct validity of the PsyCap scales, we examine the first-order factor where all psychological capabilities are correlated. The results revealed a poor fit in some indices, χ^2^(246) = 490.980, ρ < 0.001; TLI = 0.806; CFI = 0.827; GFI = 0.918; SRMR = 0.056; RMSEA = 0.046.

In addition, the difference between the first-order factor and second-order model was significant, with a Δχ^2^ = 248.0, Δdf = 101; ρ < 0.01, thus supporting the existing literature that considers that PsyCap as a second-order construct has a stronger impact on outcomes than the four psychological capabilities separately (Avey et al., 2011). Beyond these statistical differences, the option for the second-order model was made due to the fact that the second-order model makes more sense theoretically, and in light of the literature on PsyCap, while admitting that these two (second-order model and correlated factor model) will each produce similar fits in many situations, as demonstrated by Rego et al. (2012).

We analyzed the structural validity of psychological capabilities in order to test their factorial independence. Therefore a single-factor model composed of the four psychological capabilities was performed. The results show that the single-factor model revealed a very poor fit to the data, χ^2^(238) = 424.530, ρ < 0.001; TLI = 0.847; CFI = 0.654; GFI = 0.654; SRMR = 0.104; RMSEA = 0.101 (Hu and Bentler, 1999). These results support that the difference between the psychological capital as a second-order factor and the single-factor model composed of the four psychological capabilities was significant, with a Δχ^2^ = 181.5, Δdf = 93; ρ < 0.01, thus validating that the four psychological capabilities are independent constructs (Luthans et al., 2007).

The confirmatory factor analysis for the internal learning in teams scale showed adequate values. The model presents moderate and good factorial weights (λ ≥ 0.40) and appropriate individual reliabilities (*r*^2^ ≥ 0.16). After small error correlations within items, suggested by the modification indices (concretely, a covariation of errors between two items was suggested), an acceptable model fit was obtained, χ^2^(7) = 23.797, ρ < 0.001; TLI = 0.950; CFI = 0.977; GFI = 0.984; SRMR = 0.034; RMSEA = 0.071. Cronbach’s alpha coefficient for internal learning in teams was 0.76.

Furthermore, we assessed the construct validity of the study measures by running a CFA that included the second-order PsyCap construct and internal learning in teams. The final result presents acceptable adjustment indices, χ^2^(424) = 733.987, ρ < 0.001; TLI = 0.94; CFI = 0.90; GFI = 0.91; SRMR = 0.055; RMSEA = 0.039.

### Assessing Common Method Bias

The study is cross-sectional which means that both independent and dependent variables were collected from the same source at one moment in time. Given that the procedural remedies to alleviate concerns about common method variance (CMV) bias were adopted (Podsakoff et al., 2003). The amount of total variance explained by the common method factor was 14.83 percent, below the 25 percent that is suggested by Williams and McGonagle (2015). Thus, we argue that the common method bias is not a major concern to this study’s findings.

## Results

### Descriptive Statistics

Variables’ means, standard-deviations, Cronbach’s αs (in parentheses) and Pearson correlations are presented in Table 1. This table shows that internal consistencies were quite acceptable and the correlations between the variables were all significant.

**TABLE 1:** Means, standard deviations, and correlations between study variables.

Study variables	*M*	*SD*	1	2	3	4	5	6
(1) Psychological capital	4.77	0.56	(0.86)					
(2) Self-efficacy	4.69	0.74	0.645**	(0.70)				
(3) Optimism	5.15	0.81	0.710**	0.252**	(0.72)			
(4) Hope	4.83	0.68	0.720**	0.368**	0.320**	(0.74)		
(5) Resilience	4.41	0.90	0.781**	0.281**	0.418**	0.449**	(0.80)	
(6) Internal learning in teams	3.95	1.19	0.258**	0.196**	0.147**	0.234**	0.175**	(0.76)

*N = 480. Cronbach’s αs (in parentheses).*

**The correlation is significant at the 0.01 level (2-tailed).

### Cluster Analysis

We conducted a cluster analysis, to determine the psychological capital profiles, using the SPSS (v.24), taking into account that cluster analysis is a method widely used in the social sciences, as it allows the identification of subgroups or profiles of individuals within the population under study who share similar patterns on a set of variables (Bolin et al., 2014). In addition, we used *k*-means method, which is considered according to Steinley (2003), the traditional method of clustering that allows each individual case to be placed into a cluster with other observations with which it shares a similar score pattern.

**FIGURE 1:**
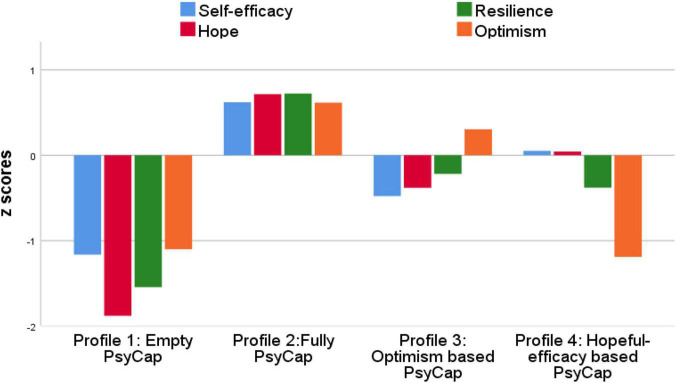
Graphical representation of the PsyCap profiles.

The cluster analysis was performed considering the four psychological capabilities: self-efficacy, optimism, hope, and resilience (Figure 1 shows the graphical representation of the profiles). The profiles were labeled as follows: Profile 1 (41 cases or 8.5% of the sample) had lower scores of all psychological capabilities: low self-efficacy, low optimism, low hope and, low resilience, reflecting lack of psychological capital, and was thus labeled Empty PsyCap. Profile 2 (195 cases or 40.6% of the sample) reflected overall psychological capital with high self-efficacy, high optimism, high hope, and high resilience, and was labeled fully PsyCap. Profile 3 (146 cases or 30.4% of the sample) showed low self-efficacy, high optimism, low hope and, low resilience, and it was labeled optimism based PsyCap. Profile 4 (98 cases or 20.5% of the sample), reflected high self-efficacy, high hope, low optimism, and low resilience and, was thus labeled hopeful-efficacy based PsyCap. Table 2 gives the mean scores of the four-cluster model (direct and normalized scores).

**TABLE 2:** Means, standard deviations, standard errors, and confidence intervals for the four-cluster model.

Profiles	*M*	*SD*	*SE*	95% confidence intervals	
				Lower bound	Upper bound
**Profile 1 (*n* = 41)** **Empty PsyCap**					
Self-efficacy	3.81 (−1.16)	0.86	0.114	−1.135	−0.688
Optimism	4.15 (−1.10)	1.02	0.097	−0.219	0.161
Hope	3.57 (−1.88)	0.50	0.082	−1.461	−1.140
Resilience	2.98 (−1.54)	0.93	0.123	−1.357	−0.874
**Profile 2 (*n* = 195)** **Fully PsyCap**					
Self-efficacy	5.12 (0.62)	0.52	0.076	0.243	0.541
Optimism	5.64 (0.62)	0.39	0.064	1.334	1.587
Hope	5.32 (0.71)	0.37	0.055	0.345	0.560
Resilience	5.04 (0.72)	0.59	0.082	0.786	1.107
**Profile 3 (*n* = 146)** **Optimism based PsyCap**					
Self-efficacy	4.35 (−0.48)	0.67	0.080	−0.534	−0.219
Optimism	5.40 (0.30)	0.40	0.068	1.087	1.354
Hope	4.50 (−0.38)	0.50	0.057	−0.479	−0.252
Resilience	4.16 (−0.22)	0.67	0.086	−0.102	0.237
**Profile 4 (*n* = 98)** **Hopeful-efficacy based PsyCap**					
Self-efficacy	4.72 (0.05)	0.57	0.062	0.242	0.540
Optimism	4.18 (−1.19)	0.58	0.052	−0.218	0.160
Hope	4.87 (0.04)	0.44	0.044	0.344	0.550
Resilience	4.10 (−0.38)	0.65	0.067	−0.101	0.236

*Measurement scale of psychological capabilities (1–6).*

*Normalized mean scores (in parentheses).*

To assess the overall fit of the four-cluster model, we first performed a multiple discriminant analysis. The results show that Wilk’s Lambda for the first discriminant function yielded a significant value of 0.150 (ρ < 0.001), meaning that the discrimination is valid. Additionally, as pointed out by AlKubaisi et al. (2019), we argue that the presented value of Wilk’s Lambda means that the variations between the psychological capabilities in the four-cluster model are significantly different. The classification results showed that 97.6 percent (hit ratio) of the participants were correctly classified into each of the four groups.

A MANOVA was carried out to analyze the contribution of the psychological capabilities making up the four-cluster model, as well as the differentiation between classes. The results reinforce the adequacy of the model, showing statistically significant differences between the four classes in the four criterion variables: Self-efficacy *F*(3; 476) = 74.70; ρ < 0.001; $\eta_{\text{p}}^{2}$ = 0.32, hope *F*(3; 476) = 220.75; ρ < 0.001; $\eta_{\text{p}}^{2}$ = 0.58, optimism *F*(3; 476) = 233.97; ρ < 0.001; $\eta_{\text{p}}^{2}$ = 0.60, resilience *F*(3; 476) = 137.60; ρ < 0.001; $\eta_{\text{p}}^{2}$ = 0.46. Therefore, considering all of the statistical parameters together, we argue that the four-cluster model is valid and is the most appropriate with the data.

### Hypotheses Testing

To test the study hypotheses, we performed ANOVAs using the four-cluster model as the independent variable and internal learning in teams as the dependent variable. The results indicate that, overall, there are significant differences between the profiles regarding internal learning in teams [*F*(3; 211) = 9.032 ρ < 0.001]. Table 3 shows the results from the ANOVA comparison between the four PsyCap profiles on internal learning in teams. Additionally, we perform Levene’s test to test the homogeneity of the variances of the data. The results show it is not possible to reject the null hypotheses that the variances are homogeneous *F* (Levene’s test) = 0.376; ρ = 0.770.

**TABLE 3:** One-way ANOVA results for internal learning in teams.

ANOVA					
	Sum of squares	df	Mean square	*F*	Sig.
Between groups	36.634	3	12.211	9.032	0.000
Within groups	643.576	476	1.352		
Total	680.211	479			

Tukey’s *Post Hoc* comparison showed that Profile 2 (fully PsyCap) and Profile 1(Empty PsyCap) differ significantly on the mean of internal learning in teams (ρ < 0.001), thus, individuals with high self-efficacy, optimism, hope, and resilience tend to score higher on internal learning in teams compared to individuals with low self-efficacy, optimism, hope, and resilience. In addition, Table 4 represents the means and standard deviations of internal learning in teams for each PsyCap profile. The Table 4 shows that regarding internal learning in teams, the fully PsyCap profile had significantly higher scores compared to the empty PsyCap profile. Hypothesis 1 was supported.

**TABLE 4:** Descriptive statistics (means and standard deviations) of internal learning in teams for each PsyCap profile.

	Internal learning in teams	
	*M*	*SD*
Profile 1: Empty PsyCap	2.866	0.115
Profile 2: Fully PsyCap	4.250	0.084
Profile 3: Optimism based PsyCap	3.280	0.159
Profile 4: Hopeful-efficacy based PsyCap	3.854	0.100

*Empty PsyCap, low self-efficacy, low optimism, low hope and, low resilience; Fully PsyCap, high self-efficacy, high optimism, high hope, and high resilience; Optimism based PsyCap, low self-efficacy, high optimism, low hope and, low resilience; Hopeful-efficacy based PsyCap, high self-efficacy, high hope, low optimism, and low resilience.*

Contrary to our predictions, although the results show that Profile 4 (hopeful-efficacy based PsyCap - with two strong levels of positive psychological capabilities) have a higher average regarding internal learning in teams than Profile 3 (optimism based PsyCap - with only one positive psychological capability), these profiles do not significantly differ on the mean of internal learning in teams (ρ = 1.000). This result was based on Tukey’s *Post Hoc* comparison. Therefore, this result takes us to reject H2.

## Discussion

The aim of this study was to analyze the relationship between psychological capital profiles and internal learning in teams. This objective is in line with the interest of previous research, which consists in analyzing the development of factors that may lead to the internal learning of students. Therefore, increasing internal learning in teams allows students to overcome labor market uncertainties caused by high rates of unemployment and job insecurity (You, 2016; Geremias et al., 2020). Furthermore, this study was motivated by the existence of few studies on psychological capital profiles carried out with students.

It should be noted that the relationship between the psychological capital profiles and internal learning in teams occurs because students who were self-efficacious regarding their study, who were optimistic and hopeful about their future, who were resilient to overcome challenges showed the highest levels of learning (Siu et al., 2013). In this study, we seek to identify which PsyCap profiles of university students show better internal learning in teams outcomes, as it is a group particularly interested in obtaining learning outcomes that allow the achievement of future goals for insertion in the labor market (Datu and Valdez, 2019).

Regarding the identification of PsyCap profiles, our results confirm the existence of student profiles that differ in their psychological capital, as mentioned by Luthans and Youssef-Morgan (2017). In addition, our results demonstrate the existence of four PsyCap profiles: a profile with a low level in the four psychological capabilities (Empty PsyCap), a profile with high levels in all four psychological capabilities (Fully PsyCap), a profile with a high level only in optimism (Optimism based PsyCap), and a profile with high levels in hope and self-efficacy but low levels in all other psychological capabilities (hopeful-efficacy based PsyCap). These findings supported the idea that students may have different levels of self-efficacy, hope, optimism, and resilience (Ferradás et al., 2019).

To test the first study hypothesis, we performed the analysis of the relationship between PsyCap profiles and internal learning in teams. In general terms, our results supported the idea that students’ psychological capital profiles are linked to their internal learning in teams. Therefore, there are significant differences between participants who scored high on all four PsyCap capabilities and participants who scored low on all four PsyCap capabilities, in the way they related to internal learning in teams. In particular, the students profile with the highest scores in all psychological capabilities (Fully PsyCap), exhibited also the best internal learning in teams outcomes. Consequently, it can be deduced that students with a Fully PsyCap profile would be characterized by developing skills that allow them to face difficult times in challenging environments, considering that they look for creative ways to solve problems and, thus, seize opportunities (Chen et al., 2019; Geremias et al., 2020).

According to Carmona–Halty et al. (2018), a positive combination of the four psychological capabilities allows students to focus on defining and executing tasks that lead to academic success. Additionally, it should be noted that the results presented between the fully PsyCap profile and internal learning in teams back up the notion that the four positive psychological capabilities work together and give rise to a second-order construct - psychological capital – that allows students to overcome uncertainties and difficulties (Luthans et al., 2012; Daspit et al., 2015; Datu and Valdez, 2019). Moreover, this finding evidence that the positive combination of the four PsyCap capabilities does, in fact, relate more strongly to learning outcomes, supporting that the interaction between these psychological capabilities create a synergistic motivational effect that allows students to overcome obstacles and remain motivated to achieve goals and learning outcomes (Huang and Luthans, 2014; Harms et al., 2018).

The students profile with low levels in all psychological capabilities (Empty PsyCap), presented the lowest scores of internal learning in teams. This result seems to occur because the absence of psychological capital does not allow students to develop a great capacity to adapt to adverse circumstances due to a lack of energy to make the necessary effort to reach the defined academic goals (Luthans et al., 2012). Additionally, this finding reinforces the fact that the four psychological capabilities have a positive impact on the levels of internal learning in teams, but their absence does not make the levels of internal learning negative. Apparently, our findings are in line with previous literature, which has emphasized that psychological capabilities only negatively influence undesirable attitudes (cynicism for change, stress, anxiety, and turnover intention) and behaviors (Liu et al., 2012; Yim et al., 2017; Çelik, 2018). Given these previous conclusions, we argue that the negative combination of the four psychological capabilities does not allow for high rates of internal team learning, but it is not strong enough to provide negative levels of internal learning in teams.

In general, our results corroborate that psychological capital is a second-order construct. However, the cluster analysis carried out revealed that there are also cases in which the scores are different across the four dimensions of psychological capital (Profile 3- optimism based PsyCap and Profile 4- hopeful-efficacy based PsyCap). Profile 3 revealed a high score only on optimism and low scores on hope, self-efficacy, and resilience. This seems to happen because optimistic individuals when challenged by controllable events, tend to engage in problem-focused and primary coping strategies, showing high levels of proactivity (Lopes, 2011). For Cansino et al. (2018), student proactivity plays an important role in explaining results in the academic settings, measured by their capacity to pass the exam and obtain higher scores.

It is important to mention that the group of students with the optimism based PsyCap profile can be characterized by the development of great future expectations regarding the achievement of defined goals, but with difficulties in showing the efforts needed to achieve these goals, when circumstances demand new challenges and alternative paths (Ferradás et al., 2019). For Haynes et al. (2006), despite optimism being a positive feature, absolute optimism can be problematic, given that students may have little experience to formulate realistic expectations.

Profile 4 revealed higher scores on hope and self-efficacy and lower ones on resilience and optimism. As reported by Djourova et al. (2019), there is a specific configuration where people tend to have higher scores on hope and self-efficacy, on the one hand, and lower scores on optimism and resilience on the other. It seems that self-efficacy and hope are related to the central core of expectancies, taking into account that they are conceptualized as cognitive sets which refer to individual results or objectives, future perspective, and determine positive individual behaviors (Magaletta and Oliver, 1999). As reported by O’Sullivan (2010), hope and self-efficacy are positively correlated and may have the strongest theoretical communality.

It may not be surprising that students with high scores on hope and self-efficacy, but low scores on optimism and resilience (hopeful-efficacy based PsyCap), can be more committed to accomplishing tasks, given their ease in defining multiple alternative paths to bypass possible obstacles (Motlagh et al., 2011; Feldman and Kubota, 2015). However, this group of students may have difficulties in maintaining positive expectations when holding future events, when faced with obstacles in their internal learning in teams process (Brewer et al., 2019; Ferradás et al., 2019). Therefore, it appears that the higher scores on self-efficacy and hope that this group of students have may be related to better internal learning in teams outcomes.

The lack of a significant relationship between the profile that presents a positive combination between self-efficacy and hope and the profile that presents optimism as the only positive capability in the way they related to internal learning in teams was not expected, which led us to reject the second hypothesis. This seems to happen because, according to Huang and Luthans (2014), only the interaction between all psychological capabilities creates a synergistic motivational effect and allows students to overcome obstacles and remain motivated to achieve goals and academic success.

Apparently, this result suggests that the existence of a psychological capability or the positive combination of two psychological capabilities (regardless of which) alone is not an effective resource for students to achieve significant internal learning in teams outcomes. As pointed out in previous studies, achieving the learning process may then require a positive combination of all psychological capabilities (Datu and Valdez, 2019; Geremias et al., 2020, 2021). Therefore, our results seem to follow this line, showing that a positive combination of the four psychological capabilities is related to a higher rate of internal learning in teams.

### Limitations and Future Directions

The present study has some limitations. First, we used quantitative method to analyze the relationship between psychological capital profiles and individual learning in teams. However, it would be important to have a counter-perspective from the qualitative analysis point of view, taking into account that previous research (e.g., Elo and Kyngäs, 2008) indicates that the potential differences between the individuals’ behaviors and mindsets related to each identified profile, cannot be analyzed based only on quantitative results. Further studies are necessary in order to explore this alternative path.

Second, we collected data at the same time, which is why it is considered a cross-sectional study. For Fitzmaurice and Ravichandran (2008), the cross-sectional study has some limitations related to the analysis of the causality of relationships between variables. Therefore, it would be interesting to have a longitudinal perspective of psychological capital profiles. According to Djourova et al. (2019), the study of psychological capital profiles using longitudinal data with at least two-time points, allows analyzing whether the profiles can yield changes over time. Thus, we argue that future studies should address this issue.

Third, the composition of the sample was very varied in terms of the typology of courses that students were taking. In this case, the configuration of the different courses may have influenced the composition of the identified psychological capital profiles, as well as the relationship of these profiles with internal learning in teams. For Song et al. (2014), learning in teams may be influenced by the variety of information and the nature of the topics under discussion. Thus, we argue that future studies should consider samples with more balanced affinities in areas of knowledge.

Fourth, as mentioned by Ferradás et al. (2019), the use of self-report scales as the only form of data collection process could negatively affect the veracity of the information received. Therefore, it may be important to combine different data collection methods. For example, conducting interviews of some students who belong to the fully PsyCap profile and some students who belong to the empty PsyCap profile might provide another perspective of analysis. Therefore, further studies are necessary in order to explore this alternative path.

Finally, it would be important to perform a similar study with a heterogeneous sample in professional settings. It is known that as knowledge and learning process has grown at a rapid pace, undergraduate students have also increased the opportunity to shape more effective educational practices (Darling-Hammond et al., 2019), therefore we argued that this fact might be a constraint for the generalizability of this study.

### Theoretical and Practical Implications

The studys’s first contribution stems from the application of a cluster analysis methodology in understanding how different psychological capital profiles foster individual learning in teams. The psychological capital profiles with the highest scores in self-efficacy, optimism, hope, and resilience (Profile 2-Fully PsyCap) exhibited also the highest scores of internal learning in teams. Establishing this linkage reinforces the role of psychological capital on academic achievement, grounded in students’ learning-motivation, the appropriateness of their study skills to particular study requirements, and their ability to earn satisfactory grades (Hazan Liran and Miller, 2017).

The second contribution seems to point that the psychological capital profile with the lowest levels of self-efficacy, optimism, hope, and resilience (Profile 1- Empty PsyCap), also had the lowest levels of internal learning in teams. These findings show the impact of having or not having positive psychological capabilities on internal learning in teams; an insight not previously revealed in the learning literature. Given this, students and teachers should consider training programs that contribute to the development of psychological capital on the learning process. Authors such as Hannah and Avolio (2010) identified, based on constructivism theory, that the development of psychological capital is related to the readiness and motivation of individuals and is composed of factors such as interest, orientation for learning, and achievement of goals.

Our third contribution is related to the lack of attention given to the study of psychological capital profiles outside the working setting, using students as a sample. Therefore, this study fills an important gap in the PsyCap field, which has led certain authors, such as Luthans and Youssef-Morgan (2017), to highlight the importance of analyzing psychological capital profiles in different contexts. Additionally, Dawkins et al. (2013) pointed out that PsyCap studies have been neglecting the importance of examining an individuals’ Psycap profile in different environments. Given this, teachers, students and other stakeholders in the learning process may pay more attention to finding out the PsyCap profiles that most contribute to internal learning in teams.

Fourth, our findings show that the profile which highlights the optimism from the remaining psychological capabilities (Profile 3- optimism based PsyCap) seems to be contributing to internal learning in teams, even when scores were lower on the other three psychological capabilities. This fact reinforces that optimistic individuals often exhibit positive behaviors that influence the way in which they face past, present, and future events in life. Optimistic individuals are more positive about events in daily life and have high levels of physical/mental well-being, and tend to use more appropriate coping strategies (Scheier and Carver, 1985; Conversano et al., 2010). Therefore, students and teachers should pay greater attention to optimism in psychological capital development programs. According to Akhoundi and Sheibani (2017), each psychological capability may be developed through concrete interventions.

Finally, it is important to mention that, based on the role of psychological capital profiles in internal learning in teams, teachers may promote the creation of a positive classroom climate that encourages the development of different psychological capital profiles that most contribute to enhancing internal learning in teams. For example, teachers should focus more on designing programs with less rigid structures that allow for greater student engagement in the search for technical content to achieve defined goals, as suggested by Carmona–Halty et al. (2018). In addition, the results of this study suggest that teachers should be aware that the development of psychological capabilities takes place when students have greater autonomy in carrying out tasks while benefiting from monitoring and support during the learning process (Luthans and Youssef-Morgan, 2017). This is a significant result that complements the current literature on possible ways to improve internal learning in teams.

## Conclusion

The aim of this study was to analyze the relationship between psychological capital profiles and internal learning in teams. Knowing further about how PsyCap profiles can differentially affect learning outcomes is crucial given that allowing students to overcome uncertainty related to academic success (You, 2016; Bouckenooghe et al., 2018; Djourova et al., 2019; Ferradás et al., 2019). For Goertzen and Whitaker (2015), studies on psychological capital are relevant because they help students to find prosperous paths even in adversity times.

The results of the study show that student profile with the highest scores in self-efficacy, optimism, hope, and resilience (Profile 2-Fully PsyCap) exhibited also the highest scores of internal learning in teams. On the other hand, as expected the student profile with the lowest scores in self-efficacy, optimism, hope, and resilience (Profile 1- Empty PsyCap) presented the lowest scores of internal learning in teams. Moreover, we also found evidence of differences toward the four psychological capital dimensions (Profile 3- optimism based PsyCap and Profile 4- hopeful-efficacy based PsyCap).

## Data Availability Statement

The raw data supporting the conclusions of this article will be made available by the authors, without undue reservation.

## Ethics Statement

The studies involving human participants were reviewed and approved by higher education institutions in the province of Huíla, Angola. The patients/participants provided their written informed consent to participate in this study.

## Author Contributions

RG designed, prepared, carried out the data collection process, and written the article. ML revised the section of the analysis and corrected the entire article. AS analyzed the data in this article. All authors contributed to the article and approved the submitted version.

## Conflict of Interest

The authors declare that the research was conducted in the absence of any commercial or financial relationships that could be construed as a potential conflict of interest.

## Publisher’s Note

All claims expressed in this article are solely those of the authors and do not necessarily represent those of their affiliated organizations, or those of the publisher, the editors and the reviewers. Any product that may be evaluated in this article, or claim that may be made by its manufacturer, is not guaranteed or endorsed by the publisher.
